# Exploring the Enigmatic Link: Unraveling the Relationship Between Obesity and Cigarette Smoking Among Diverse College Students at Imam Mohammed Ibn Saud Islamic University in Riyadh, Saudi Arabia

**DOI:** 10.7759/cureus.56158

**Published:** 2024-03-14

**Authors:** Ahmed F Alanazi, Rayan Ahmed N Alghamdi, Saad O Alhokail, Abdullah M Jailan, Abdulrahman A Aljaser, Abdulrahman Alkanhal, Khalid A Bin Abdulrahman

**Affiliations:** 1 Medicine and Surgery, Imam Mohammad Ibn Saud Islamic University, Riyadh, SAU; 2 Family and Community Medicine, Imam Mohammad Ibn Saud Islamic University, Riyadh, SAU

**Keywords:** saudi arabia, riyadh, association, smoking, obesity

## Abstract

Background: Obesity is defined as an excess of body fat. This medical condition frequently results in a high BMI and an increased risk of a variety of health problems, including diabetes, cardiovascular disease, and certain types of cancer. Cigarette smoking includes inhaling smoke created by the combustion of tobacco. It is linked to a variety of health issues, including lung cancer, heart disease, and respiratory ailments, and is a primary cause of preventable disease and premature death worldwide. The association between obesity and cigarette smoking is complex and incompletely understood. This study aims to investigate the intriguing association between obesity and cigarette smoking among diverse college students at Imam Mohammed Ibn Saud Islamic University in Riyadh, Saudi Arabia.

Methodology: The study was conducted as an observational study, specifically an analytical cross-sectional study, to measure the prevalence of cigarette smoking and obesity and their association. This type of study is chosen because of its advantages including targeting a large sample in a short time and inexpensive way, with no loss to follow-up, unlike some other study designs.

Results: In this study, we were able to collect data from 603 participants, of which 57.4% were male and 67.8% of them aged between 20 and 24 years old. Moreover, we found that 39.6% had normal weight; however, the prevalence of obesity, overweight, and underweight were 24%, 28.1%, and 8.3%, respectively. Considering the prevalence of smoking, we found that 22.6% of the participants reported being current smokers, while 5.3% were former smokers. There is a significant difference between participants with different BMIs (P=0.001). The prevalence of smoking was significantly higher in obese and overweighted participants (35.1% and 31.3%, respectively) compared with 28.4% in normal-weighted participants.

Conclusion: The prevalence of smoking and obesity in this study was significantly higher than reported in different studies. Moreover, we found a significant relationship between smoking and obesity; however, further investigation should be conducted to determine the cause of this relationship.

## Introduction

Overweight and obesity are defined as an excessive buildup of fat in adipose tissue, which results from impairment of physical activity and psychological health [1]. Overweight and obesity are regarded as serious public health issues in many countries across the world. Obese individuals have a greater risk of all-cause mortality, as well as several serious health problems and diseases such as type 2 diabetes, hypertension, dyslipidemia, coronary heart disease, stroke, obstructive sleep apnea, cancers, and breathing complications, as well as difficulty with physical functioning and poor quality of life [2-5]. Overweight and obesity affect more than 1.9 billion and 650 million persons globally aged 18 and up, respectively, and the number of deaths associated with overweight and obesity is higher than that linked to underweight [1]. The recommended way to categorize obesity is BMI; a BMI of 25-30 is considered overweight, whereas a BMI of more than 30 is obese [6].

In the report discussing obesity and eating habits, it is noted that there has been a rapid socio-cultural change in the Arab region due to its expanding economy, and this change has had an impact on eating habits, which has subsequently led to an increase in the number of overweight and obese individuals among Saudis [7]. Obesity can be caused by other factors such as psychological stress, and a great example would be university life and the environment [5]. Efforts are required to alleviate the intense academic pressure experienced by college students, taking into account how factors such as gender, year in school, and type of college may influence this stress. These interventions could also help in preventing overweight and obesity among college students [8].

Excess weight is linked to health risks, and habits such as smoking and maintaining good sleep habits also play a role in this association [9]. There is a notable prevalence of obesity and overweight among students in the Healthcare Science College, underscoring the importance of promoting healthy lifestyle choices, nutritious eating habits, and regular physical activity to prevent complications associated with obesity [10]. Men had a higher prevalence of overweight, while obesity was more common among women. The high prevalence of overweight and obesity among young adults aged 18-29 years is a significant public health concern [11]. Despite the fact that obesity causes a lot of health issues, there are only a few studies that studied the relationship between obesity and cigarette smoking. Our aim in this study is to investigate the association between obesity and smoking.

Primary objective: To investigate the relationship between cigarette smoking and body weight status among college students at Imam Mohammed Ibn Saud Islamic University, Riyadh, Saudi Arabia.

Secondary objective: To assess the prevalence of smoking and obesity among students at Imam Mohammed Ibn Saud Islamic University, Riyadh, Saudi Arabia.

## Materials and methods

Study design

This study employed an observational research design, specifically an analytical cross-sectional study, conducted in November 2021 at Al-Imam Mohammed Ibn Saud Islamic University in Riyadh. The primary objective of the study was to determine the prevalence of cigarette smoking and obesity among university students and to investigate the potential association between these two variables. By adopting this design, the researchers aimed to gather comprehensive data regarding the current state of smoking and obesity within the student population and explore any potential relationships between these factors.

Study population

The population of this study is Al-Imam Mohammed Bin Saud University students. The selection of subjects was through a convenience sampling method. Regarding the eligibility criteria, all students in this university who responded to the questionnaire were included in this study, regardless of which college they belong. However, there were no exclusion criteria in this study.

Sample size

The sample size was 603 students (57.4% male and 42.6% female), and 67.8% of them aged between 20 and 24 years old. The sample size was chosen to limit the risk of error and to make the results more valid to be interpreted.

Data collection method

An e-questioner was sent to the students through email and social media applications, and subjects responded to it. Data collection tools included BMI to detect obesity, by asking the subjects about their weight and height. Obesity is BMI >30 kg/m^2^. Cigarette smoking was detected by asking about the smoking status, amount, frequency, and duration [8]. The questionnaire was developed based on a literature review, which comprised 32 questions. It includes three sections: socio-demographic, weight status, and smoking status. It was conducted in Arabic to gather as much valid data as possible.

Variables and measurements

Obesity: It is an independent variable and is defined as a BMI of >30 kg/m^2^. BMI was derived from weight and height: weight (kg) / (height (m) x height (m)).

Smoking behavior: It is an independent variable that was recorded via a self-administered questionnaire. Information collected included the following: current smoking status, amount smoked, duration of smoking, and time since quitting smoking. Smoking status was categorized into current, former, or never smoker. The amount smoked by current smokers was assessed in three ways: number of cigarettes smoked (per day), duration of smoking (in years), and lifetime consumption of cigarettes (pack years). Participants provided information on the first two pack-years were derived from (number of cigarettes smoked (per day) x duration of smoking (in years)) / 20. The number of cigarettes smoked per day was used to categorize current smokers into heavy (>20 cigarettes per day), moderate (10-20 cigarettes per day), and light (<10 cigarettes per day) smokers [8].

The amount previously smoked by former smokers was assessed in two ways: the amount smoked and the duration of smoking (in years). In relation to the amount smoked, former smokers were asked whether they had smoked on most/all days or were occasional smokers. The latter were then asked whether they had smoked at least 100 cigarettes over their lifetime or not. These two variables were combined to classify former smokers into heavy (smoked most/all days), moderate (occasional smoker who smoked at least 100 cigarettes in total), and light (occasional smoker who smoked less than 100 cigarettes in total) former smokers. Both former and current smokers were included in the analyses of time (in years) since quitting with time recorded as 0 for current smokers. Other covariants were included as GPA, age, gender, marital status, financial status (monthly income), and type of cigarette used.

Statistical analysis

After collecting the data, MS Excel was used for data entry, while Statistical Product and Service Solutions (SPSS, version 26; IBM SPSS Statistics for Windows, Armonk, NY) was used for data analysis. Descriptive analysis, including frequency and percent, was used for the description of categorical variables. Chi-test was used to determine the relation between smoking and the prevalence of obesity where all statement was considered significant if the p value was lower or equal to 0.05.

## Results

In this study, we were able to collect data from 603 participants, of which 57.4% were male and 67.8% of them aged between 20 and 24 years old. Considering residency, we found that most of the participants reported living with family, and 84.6% were single. Moreover, 19.2% of the participants were in the third level, 16.6% were in the first level, and 15.6% were at level five. Moreover, 21.4% of the participants reported having other jobs other than in a university as students (Table 1).

**Table 1:** Demographic factors of the participants (N=603)

		Count	N %
Gender	Male	346	57.4%
	Female	257	42.6%
Age	19 years or less	76	12.6%
	20-24	409	67.8%
	25-29	44	7.3%
	30 or older	74	12.3%
Residency	With family	515	85.4%
	Rent apartment	42	7.0%
	With college	32	5.3%
	Other	14	2.3%
Marital status	Single	510	84.6%
	Married	79	13.1%
	Divorced	11	1.8%
	Widow	3	0.5%
Level	First	100	16.6%
	Second	25	4.1%
	Third	116	19.2%
	Fourth	40	6.6%
	Fifth	94	15.6%
	Six	82	13.6%
	Other	146	24.2%
Having a job other than in a university	Yes	129	21.4%
	No	474	78.6%

**Figure 1:**
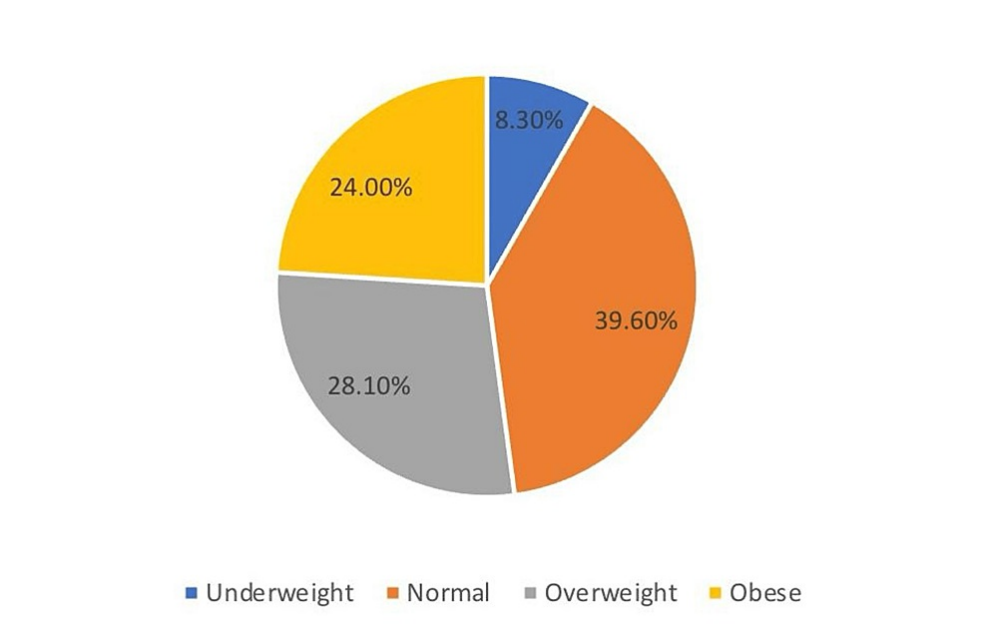
The frequency of BMI subscales

Moreover, the BMI was calculated for all participants, and we found that 39.6% had normal weight. However, the prevalence of obesity, overweight, and underweight was 24%, 28.1%, and 8.3%, respectively (Figure 1).

Considering the prevalence of smoking, we found that 22.6% of the participants reported being current smokers, while 5.3% were former smokers. Furthermore, we found that 51.2% of smokers reported using e-cigarettes, and 40.5% reported smoking shisha. Most of the smokers reported starting smoking at the age of 16-18 years (37.5%) and 18-22 years (27.4%). Moreover, 49.4% of the smokers reported having the last cigarette on the last day, and 36.3% of them consumed 6-10 cigarettes each day. Furthermore, 37.5% of the smokers reported smoking more in the first few hours after waking up than the rest of the day and 51.8% smoking in school and university at first. Reasons for smoking included inducing calmness and relaxation (85.7%), reducing anxiety rate (78.6%), being an act of courage (63.7%), and a different taste in the mouth (60.1%) (Table 2). 

**Table 2:** The prevalence of smoking among the samples and characteristics of smoking

		Count	N %
Smoking	Yes	136	22.6%
	No	435	72.1%
	Former smoker	32	5.3%
Age of starting smoking	7 years or less	5	3.0%
	8-11	23	13.7%
	12-15	31	18.5%
	16-18	63	37.5%
	18-22	46	27.4%
Last time of smoking	Last one or two days	83	49.4%
	During the last week	43	25.6%
	2 weeks to a month ago	23	13.7%
	Months ago	11	6.5%
	More than one year	8	4.8%
How many cigarettes do you smoke each day?	Less than 5	48	28.6%
	6-10	61	36.3%
	11-15	27	16.1%
	16-20	27	16.1%
	More than 20	5	3.0%
Do you smoke more in the first few hours after waking up than the rest of the day?	Yes	63	37.5%
	No	105	62.5%
When you first smoked, where did you usually smoke?	school or university	87	51.8%
	At friends’ home	28	16.7%
	Public setting	31	18.5%
	Home	12	7.1%
	Other	10	6.0%
Do you feel a different taste in your mouth when you smoke?	Yes	101	60.1%
	No	67	39.9%
Do you feel calm and relaxed when smoking?	Yes	144	85.7%
	No	24	14.3%
Does your anxiety rate decrease when you smoke?	Yes	132	78.6%
	No	36	21.4%
Did you think smoking was cool or out of courage during the early stages of smoking?	Yes	107	63.7%
	No	61	36.3%

Among smokers, 29.3% of them thought that obesity is not a medical condition, and 40.9% of the smokers reported having knowledge about BMI. Moreover, 44.1% of the participants reported knowing the definition from self-estimation, while 41.2% depended on scientific studies. Furthermore, 45% of the smokers reported having difficulties deciding what to eat, while 73.1% of them reported that they care about their appearance. Considering eating habits, we found that 40.4% of the smokers would have breakfast three to four days per week, while 19.9% would consume sweets more than six times in the last seven days. Moreover, 62.6% of smokers knew that smoking is a risk factor for obesity complications, and 40.1% thought that, once you reach your ideal weight, you can stop your healthy habits, and 78.5% thought that healthy people eat healthy and unhealthy foods (Table 3).

**Table 3:** Smokers’ knowledge and attitude toward the definition of obesity

		Count	N %
Do you think obesity is a medical condition?	Yes	118	70.7%
	No	49	29.3%
Do you know the recommended body mass index (BMI) from the World Health Organization?	Yes	70	40.9%
	No	101	59.1%
What is your definition of obesity?	A definition based on scientific studies	70	41.2%
	Self-definition	75	44.1%
	Other	25	14.7%
Do you have a hard time deciding what to eat?	Yes	77	45.0%
	No	94	55.0%
Do you care with your appearance	Yes	125	73.1%
	No	46	26.9%
How many times do you eat breakfast per week?	No breakfast	11	6.4%
	Everyday	42	24.6%
	1-2 days/week	49	28.7%
	3-4 days/week	69	40.4%
How many sweets (chocolate, pastry, etc.) have you eaten in the past seven days?	Nothing	17	9.9%
	1-2	40	23.4%
	3-4	36	21.1%
	5-6	44	25.7%
	More than 6	34	19.9%
Do you think smoking is a risk factor for obesity complications?	Yes	107	62.6%
	No	64	37.4%
Do you think that once you reach your ideal weight you can stop your healthy habits?	Yes	65	40.1%
	No	97	59.9%
Can healthy people eat healthy and unhealthy foods?	Yes	135	78.5%
	No	37	21.5%

In Table 4, we represented the relation between BMI subdivisions and smoking prevalence finding that there is a significant difference between participants with different BMIs (P=0.001). The prevalence of smoking was significantly higher in obese and overweighted participants (35.1% and 31.3%, respectively) compared with 28.4% in normal-weighted participants. Moreover, we found that obese and overweight participants tend to smoke at an earlier age or start smoking at an earlier age, which is associated with an increased incidence of obesity (P=0.002).

**Table 4:** The relation between BMI and smoking prevalence and characteristics * Significant difference

		BMI				P-value
		Underweight	Normal	Overweight	Obese	
Smoking	Yes	5.2%	28.4%	31.3%	35.1%	0.001*
	No	9.2%	44.5%	26.1%	20.2%	
	Former smoker	9.4%	21.9%	40.6%	28.1%	
Do you smoke more in the first few hours after waking up than the rest of the day?	Yes	6.6%	26.2%	44.3%	23.0%	0.072
	No	5.7%	27.6%	26.7%	40.0%	
Age of starting smoking	7 years or less	0.0%	20.0%	0.0%	80.0%	0.002*
	8-11	13.0%	26.1%	30.4%	30.4%	
	12-15	6.5%	35.5%	22.6%	35.5%	
	16-18	6.6%	21.3%	54.1%	18.0%	
	18-22	2.2%	30.4%	17.4%	50.0%	

## Discussion

In the study of Dare et al., the authors reported that smoking is related to less prevalence of obesity; however, the same study found that former smokers were more likely to be obese than non-smokers and current smokers [12]. The aim of this study was to assess the prevalence of smoking and obesity among students at Imam University, Saudi Arabia, and discuss the relationship between smoking and obesity. According to our study, the prevalence of obesity and overweight among students was 24% and 28.1%, respectively. In a previous study conducted by Ginawi et al., the prevalence of obesity among the public population in the Hail region, Saudi Arabia, was 36.9% [13]. In another study conducted by Peltzer et al., among university students, the authors reported a prevalence of obesity and overweight of 5.2% and 14.1%, respectively [14]. In Sohag University, Egypt, the prevalence of obesity and overweight among students was 38.5% [15]. The difference in the reported prevalence of obesity among studies is due to the different settings of the study where many previous studies reported that modern life in Saudi Arabia is an accouraging type of life of obesity due to the increase in the possibility of fast food and increasing time of staying at home, especially after the COVID-19 pandemic [7,16,17]. Another reason was the different methods of determining obesity, and in our study, it depended on the calculated BMI using the reported height and weight of students.

Moreover, we found a prevalence of current smoking among students of 22.6%, while 5.3% reported being former smokers. In the study of Amin et al., the authors found a prevalence of smoking among students (11%) at Kafr El Sheikh University, Egypt [18], while in another study conducted at Hodeidah University, Yemen, Nasser et al. found a prevalence of 33.1% [1]. Among Malaysian university students, Al-Naggar et al. reported a prevalence of 29% [19].

Considering the relationship between smoking and obesity, we found a significant relationship between the incidence of obesity and the prevalence of smoking among students where the prevalence of smoking among obese participants was 35.1% compared with 28.4% in normal-weight participants. Our results are in agreement with the results of Reas et al., who found that the prevalence of obesity was higher in smokers than in non-smokers. However, the study found that former smokers gained 3-5 kg higher than current smokers [20]. The same results were reported in the study of Ginawi et al., who reported the prevalence of obesity in current smokers, former smokers, and non-smokers was 24.9%, 45.0%, and 27.6%, respectively [13]. The increased prevalence of obesity in former smokers and the low prevalence of obesity in current smokers were reported in different studies [21-24]. Moreover, another study showed that there is no significant difference between current smokers, smokers, and non-smokers in BMI [25]. These results indicated that there is a significant relationship between obesity and smoking, which was reported in many previous studies [26-29].

This study had certain limitations that should be acknowledged. Firstly, it relied on self-reported questionnaires, including clinical information, which can introduce personal bias. Some participants may not be completely honest in reporting their information, particularly when it comes to sensitive topics such as smoking. Additionally, there may be variations in how participants report their physical characteristics, such as weight and height. To address this, it is recommended to collect data through clinical presentation, which unfortunately was challenging in this study due to the COVID-19 situation. Another limitation is the potential for recall bias, as some questions in the questionnaire required participants to remember past information. Another notable limitation is the nonresponse rate in the questionnaire. Despite efforts to maximize participant response rates, a certain percentage of individuals included in the sample did not complete the questionnaire. This nonresponse introduces the possibility of bias, as those who chose not to respond may differ from those who did respond in systematic ways. As a result, the generalizability of the study's findings to the broader population may be limited. To improve the representativeness of future studies, it is important to explore strategies to mitigate nonresponse and enhance participation rates. Furthermore, this study utilized a convenience sampling method, which involves recruiting readily available and willing participants. However, this approach can introduce selection bias and limit the external validity of the findings. The sample obtained through convenience sampling may not accurately represent the larger population of interest. Therefore, it is advisable for future research to employ more rigorous sampling methods such as random sampling or stratified sampling to enhance the generalizability of the results.

## Conclusions

In summary, our study revealed a worrisome finding: the rates of smoking and obesity observed in our research surpassed those documented in previous studies. However, it is important to acknowledge that this is a single cross-sectional study, and caution should be exercised when making conclusions about broader trends. This finding not only raises alarms but also underscores the urgency for proactive interventions. Furthermore, our analysis illuminated a noteworthy correlation between smoking and obesity, suggesting a potential interplay between these two health risk factors. However, delving deeper into the underlying mechanisms driving this relationship is imperative. Thus, future investigations must prioritize unraveling the intricate web of causation, which may hold the key to developing targeted strategies aimed at curbing these detrimental habits and fostering healthier lifestyles.
